# Rapid viral rebound after analytical treatment interruption in patients with very small HIV reservoir and minimal on‐going viral transcription

**DOI:** 10.1002/jia2.25453

**Published:** 2020-02-27

**Authors:** Pieter Pannus, Sofie Rutsaert, Stéphane De Wit, Sabine D Allard, Guido Vanham, Basiel Cole, Coca Nescoi, Joeri Aerts, Ward De Spiegelaere, Achilleas Tsoumanis, Marie‐Madeleine Couttenye, Natacha Herssens, Marie‐Angélique De Scheerder, Linos Vandekerckhove, Eric Florence

**Affiliations:** ^1^ Departments of Clinical and Biomedical Sciences Institute of Tropical Medicine Antwerp Belgium; ^2^ Department of Biomedical Sciences University of Antwerp Antwerp Belgium; ^3^ Department of General Internal Medicine HIV Cure Research Centre Ghent University Hospital and Ghent University Ghent Belgium; ^4^ Saint Pierre University Hospital Université Libre de Bruxelles Brussels Belgium; ^5^ HIV Reference Centre Universitair Ziekenhuis Brussel Brussels Belgium; ^6^ Vrije Universiteit Brussel Brussels Belgium; ^7^ Department of Morphology Faculty of Veterinary Medicine Ghent University Ghent Belgium; ^8^ Antwerp University Hospital University of Antwerp Antwerp Belgium

**Keywords:** HIV, post‐treatment control, treatment interruption, viral reservoir, total HIV DNA, cell‐associated HIV RNA

## Abstract

**Introduction:**

Viral remission after analytical treatment interruption (ATI), termed post‐treatment control, has been described in a small proportion of HIV‐positive patients. This phenomenon has been separately associated to both low levels of HIV‐1 proviral DNA as well as cell‐associated RNA. We investigated whether the combination of both parameters could help predict delayed viral rebound after treatment interruption (TI).

**Methods:**

We conducted an open single‐arm ATI study in four Belgian HIV reference centres from January 2016 to July 2018. Eligible participants were adults who had fewer than 50 HIV‐1 RNA copies/mL for more than two years, more than 500 CD4 cells/µL for more than three months, and were in general good health. Consenting participants who had fewer than 66 copies total HIV‐1 DNA (t‐DNA) and fewer than 10 copies cell‐associated HIV‐1 unspliced RNA (US‐RNA) per million peripheral blood mononuclear cells (PBMCs), interrupted therapy and were monitored closely. Antiretroviral therapy (ART) was resumed after two consecutive viral loads exceeding 1000 copies or one exceeding 10,000 copies/mL. The primary outcome was the proportion of participants with fewer than 50 HIV‐1 RNA copies/mL 48 weeks after TI. Secondary outcomes were time to viral rebound, the frequency of serious adverse events (AEs) and evolution of t‐DNA and US‐RNA after TI.

**Results:**

All 16 consenting participants who interrupted therapy experienced rapid viral rebound two to eight weeks after TI. No serious AEs were observed. Levels of t‐DNA and US‐RNA increased after TI but returned to pre‐ATI levels after treatment restart. None of the studied demographic, clinical and biological parameters were predictive of time of viral rebound.

**Conclusions:**

The combination of low levels of t‐DNA and US‐RNA in PBMCs, corresponding respectively to a small and transcriptionally silent viral reservoir, is not predictive of viral remission after TI in patients on ART.

## 1 Introduction

Although antiretroviral therapy (ART) effectively blocks HIV replication, HIV persists in a reservoir of latently infected CD4+ T cells 1. This causes most patients to experience viral rebound within weeks after treatment interruption 2. Viral remission after analytical treatment interruption (ATI), termed post‐treatment control (PTC), has been described in a small proportion of patients 3, 4, 5. Although PTC has been associated with early treatment initiation 6, we and others showed that some PTCs initiated ART in the chronic phase 4, 7. A single common characteristic of all well‐documented PTCs is the presence of a very small reservoir similar to “elite” controllers 8, 9, 10, 11, 12.

Besides the size of the viral reservoir (measured as total HIV‐1 cell‐associated DNA or t‐DNA), its transcriptional activity (measured as HIV‐1 cell‐associated unspliced RNA or US‐RNA) also independently correlates with the delay of viral rebound after ATI 9, 11, 13. Indeed, a pooled analysis of six AIDS Clinical Trials Group ATI studies aiming at identifying predictors of viral rebound, revealed that higher levels of US‐RNA are associated with shorter time to viral rebound (TTVR) after ATI 11. However, no prospective studies have investigated the association between TTVR and the combination of a very small latent viral reservoir (t‐DNA) and minimal on‐going viral transcription (US‐RNA) before ATI.

We therefore designed a single‐arm open ATI trial where patients on ART were selected based on both low levels of t‐DNA and US‐RNA. The aim of the study was to evaluate whether these combined selection criteria could help to better predict delayed viral rebound.

The primary objective of this study was to determine the proportion of participants with plasma viral load (pVL) < 50 copies/mL at 48 weeks after ATI. Secondary objectives included a safety assessment of the ATI, TTVR, kinetics of pVL as well as evolution of viral reservoir t‐DNA and US‐RNA during ATI and after restarting ART. Exploratory objectives included the identification of other potential factors predictive of TTVR, such as demographic, clinical and biological parameters, including CD4+ T cell nadir, ultrasensitive plasma viral load (usVL) 14, integrated DNA and HIV‐1 cell‐associated long terminal repeat (LTR)‐RNA levels 15 as well as *in vitro* assays for viral RNA release 16 and infectious viral outgrowth 17. Finally, we investigated the origin of rebound viruses by comparing *in vivo* rebound with *in vitro* outgrowth viruses by single genome analysis (SGA).

## 2 Methods

### 2.1 Study design and participants

The study ran from 25 January 2016 to 22 July 2018. In the first stage, 114 consenting individuals were recruited from a total patient population of 7212 HIV‐1–positive people on ART from four major Belgian HIV Reference Centers: one in Antwerp (Institute of Tropical Medicine (ITM)), two in Brussels (University Medical Center (UMC) and Universitair Ziekenhuis Brussel (UZB)) and one in Ghent (Universitair Ziekenhuis Gent (UZG)). Inclusion criteria included nadir CD4+ T‐cell count >300 cells/µL, current CD4+ T‐cell count ≥500 cells/µL for ≥3 months and pVL < 50 copies/mL for ≥2 years. Exclusion criteria included, among others, pregnancy, breastfeeding, active hepatitis B or C infection and any psychiatric or psychological disorder that could interfere with participation in an ATI study.

Stage 1 participants were screened for t‐DNA and US‐RNA levels. Participants with t‐DNA < 66 copies per million peripheral blood mononuclear cells (PBMCs) and US‐RNA < 10 copies per million PBMCs were eligible for stage 2. We have not included a control arm of participants with a high viral reservoir mainly for futility and safety reasons. The threshold for t‐DNA was based on the DNA levels from a previous clinical study 18. The 15th percentile of the cohort was used as a threshold to include only patients with a small reservoir. The threshold for US‐RNA corresponded to the limit of detection of the assay when performed in three replicate reactions.

Consenting participants eligible for stage 2 were psychologically examined to evaluate their motivation for participation and to avoid potential misconceptions on study objectives 19. A leukapheresis was performed and ART was interrupted (baseline) after sexual counselling. Participants were followed for 48 weeks or until ART resumption criteria were reached. In line with other treatment interruption studies 5, these criteria included two consecutive viral loads >1000 copies/mL measured at least three days apart, a single viral load >10,000 copies/mL, CD4+ T‐cell count drop to <350 cells/µL at two consecutive measurements at least two weeks apart or a drop of more than 50% compared to baseline, any new significant clinical condition, a new sexually transmitted infection, pregnancy or participant withdrawal from the study.

**Figure 1:**
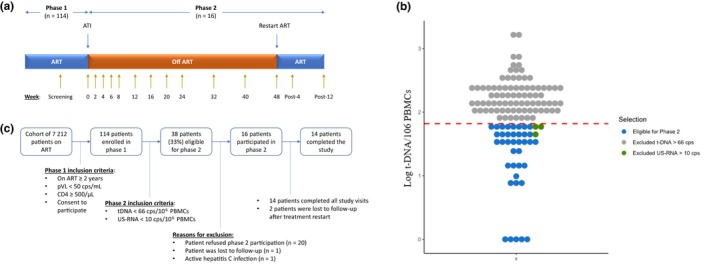
**(a)** Overview of stage 1 and 2 of the study. Timepoints at which blood was drawn for plasma and PBMC separation are indicated with yellow arrows. ART is restarted when treatment resumption criteria are reached or 48 weeks after treatment interruption. **(b)** t‐DNA values in copies per million PBMCs for all stage 1 participants. Participants with t‐DNA values >66 copies per million PBMCs are indicated with grey dots (n = 75). Patients with t‐DNA values below 66 copies but US‐RNA values above 10 copies per million PBMCs are indicated with green dots (n = 3). Patients with t‐DNA and US‐RNA values below 66 and 10 copies per million PBMCs respectively are indicated with blue dots (n = 38) and are eligible for stage 2 participation. **(c)** Flow chart with number of patients screened until number of patients who completed the study. ART, antiretroviral therapy; ATI, analytical treatment interruption; PBMC, peripheral blood mononuclear cell; pVL, plasma viral load; t‐DNA, total HIV‐1 DNA; US‐RNA, cell‐associated HIV‐1 unspliced RNA.

On weeks 2/4/6/8/12/16/20/24/32/40/48 post ATI as well as week 4 and 12 post ART restart, whole blood was collected for plasma and PBMC separation (Figure 1a). Plasma was used for immediate pVL measurement and the remainder was stored at −80°C. All PBMCs were stored in liquid nitrogen.

### 2.2 Ethics statement

The study was approved by the internal review board of the ITM in Antwerp, the ethics committees of the University Hospitals of Antwerp (UZA), Ghent (UZG) and Brussels (UMC St. Pierre, UZB) (respective approval numbers: 1012/15, 15/31/321, 2015/0771, AK/15‐07‐84/4539 and 2015/261) and was registered on http://clinicaltrials.gov with the number NCT02590354.

### 2.3 Safety assessments

Safety assessments were performed at each study visit and included evaluation of clinical signs as well as laboratory parameters. The severity of adverse events (AEs) was scored according to the Division of AIDS toxicity table 20.

### 2.4 Cell‐associated HIV‐1 DNA and RNA measurements

At screening, t‐DNA and US‐RNA were measured by droplet digital polymerase chain reaction. Subsequently, in the selected stage 2 participants, the following HIV‐1 measurements were performed: t‐DNA and cell‐associated LTR HIV‐1 RNA (LTR‐RNA) in PBMCs at baseline, weeks 2/4/6/8/12/16/20/24/32/40/48 post‐ATI, and week 12 post‐ART restart (PW12); integrated HIV‐1 DNA in PBMCs at baseline, before ART restart and PW12; LTR‐RNA in CD4+ T cells at baseline (Figure 1a). US‐RNA targets the gag/pol region and represents a late complete transcript, whereas LTR‐RNA targets the 5′‐LTR region, representing the earliest incomplete transcript 15, 21. For technical details see Methods S1.

### 2.5 Ultra‐sensitive pVL

Ultrasensitive pVL was measured as described by Leal *et al.* with minor modifications 14. In short, 10 mL of EDTA plasma was concentrated to 0.8 mL by ultracentrifugation at 170,000× g for one hour in a swinging bucket rotor (Beckman Coulter, Brea, CA, USA) and tested on the Roche Cobas^®^ 4800 system. usVL results were multiplied by 0.08 (0.8/10) to account for the concentration. The assay can reliably detect viral loads ≥5 copies/mL.

### 2.6 Quantitative viral outgrowth assay

The quantitative viral outgrowth assay (qVOA) was performed on 24 × 10^6^ CD4+ T cells as described by Laird *et al.* with some modifications 17. For technical details see Methods S2.

### 2.7 Viral release assay

The viral release assay (VRA) was performed as described by Cillo *et al.* with minor modifications 16. PBMCs and CD4+ T cells were cultured in unstimulating and stimulating conditions. The unstimulated condition refers to overnight resting in Roswell Park Memorial Institute 10% fetal calf serum + 0.6% penicillin/streptomycin + 100 nmol/L Dapivirine + 100 nmol/L Raltegravir. For the stimulated condition, cells were incubated for seven days in the same complete medium with additional 50 ng/mL phorbol myristate acetate (Sigma, Diegem, Belgium) and 500 ng/mL ionomycin (Sigma). PBMCs were plated in six‐well plates at 15 × 10^6^ cells per well in 5 mL medium. CD4+ T cells were plated in 24‐well plates at 5 × 10^6^ cells per well in 2.5 mL medium. Supernatant was stored at −80°C until assessment of virus release by quantitative real‐time PCR with the commercial COBAS HIV‐1 viral load test (Roche, Basel, Switzerland).

### 2.8 SGA comparing viruses from qVOA and rebound plasma

Supernatant from TZM‐Bl‐positive qVOA wells from three participants (1011, 4012 and 3033) with more than two positive qVOA wells (resp. 13, 11 and 3 wells) was harvested and 10 µL was subjected to RNA extraction using the QIAamp mini viral RNA kit (Quiagen, Venlo, The Netherlands), according to the manufacturer's instructions. Viral RNA was extracted from plasma samples at the time of rebound, as described previously 22. Subsequently, cDNA was generated using the Superscript III RT kit (Invitrogen, Carlsbad, CA, USA), according to the manufacturer's instructions. In the case of rebound samples, a dilution series (1:3 to 1:243) of the cDNA was made, aiming for ≤30% positive reactions, and the V1‐V3 *env* region (894 bp) was amplified using a nested PCR, as described previously 23. For qVOA samples, five replicates of the same nested PCR were performed on bulk cDNA, to ensure the presence of only a single virus per positive well. Positivity of reactions was checked on a 1% agarose gel, and amplicons were sequenced from both ends by Sanger sequencing, using second round primers.

### 2.9 Phylogenetic analyses

Phylogenetic analyses on V1‐V3 sequences derived from qVOA wells and plasma were performed as described previously 23, 24. Sequences that did not meet quality standards and hypermutated sequences were omitted from further analysis. The remaining sequences were used to construct contigs, which were aligned with MUSCLE 25. In the case of qVOA sequences, the five replicate contigs per positive well were multiple aligned and the consensus sequence was extracted. Maximum likelihood phylogenetic trees were constructed with PhyML v3.1 26, using the general time‐reversible nucleotide substitution model with 1000 bootstraps. Additionally, a neighbour‐joining tree with all sequences from the three subjects was created to check for inter‐sample contamination.

### 2.10 Statistical analysis

Categorical data are presented as frequency counts and percentages, and continuous variables are presented as medians and interquartile ranges. Continuous measurements were compared using the Wilcoxon signed‐rank and Mann‐Whitney test in the case of dependent and independent samples respectively. Spearman's rank correlation coefficients were calculated to determine correlations between VRA and US‐RNA results. To analyse virological markers over time a Friedman Rank Sum test, followed by a *post‐hoc* Nemenyi test for unreplicated blocked data, was performed on participant samples without missing data for baseline, timepoint before restart ART and 12 weeks post‐ATI. Cox proportional hazard models were used to calculate the probability of viral rebound at each time point and to assess associations of clinical, virological and immunological parameters with TTVR dynamics.

## 3 Results

### 3.1 Participant selection and clinical characteristics

In stage 1, 114 patients who provided written informed consent were recruited (characteristics in Table S1). Median t‐DNA and US‐RNA levels were 107 [inter quartile range, IQR: 46 to 205] and 3.0 [IQR: 1.0 to 6.0] copies per million PBMCs respectively. Forty‐one (36.0%) had t‐DNA levels <66 copies per million PBMCs. Of those, 38 (92.7%) also had US‐RNA levels <10 copies per million PBMCs and were therefore eligible for treatment interruption (Figure 1b). Twenty‐two did not consent or were excluded for participation in stage 2 for various reasons (Figure 1c). The 16 remaining stage 2 participants included 15 men and one woman with median age 44 years [IQR: 38 to 54], median time on ART 4.0 years [IQR: 2.9 to 6.2] and median CD4+ T‐cell count 758 cells/µL [IQR: 679 to 845]. Time since diagnosis was significantly shorter in the 16 patients who participated in stage 2 (median [IQR]: 3.9 years [2.8 to 6.3]) as compared to the patients who were not eligible for stage 2 (median [IQR]: 6.2 years [4.0 to 9.6]). Detailed participant characteristics are given in Table 1.

**Table 1:** Individual characteristics of stage 2 study participants (n = 16)

Study ID	Time on ART (years)	Time with undetectable pVL (years)	CD4 nadir (cells/µL)
1011	3.2	2.9	446
1029	3.0	2.8	661
1038	4.7	4.2	379
1043	6.9	3.4	435
1044	11.4	7.1	328
2007	2.6	2.5	506
2022	2.6	2.5	302
2025	6.4	5.5	402
3024	15.6	8.5	467
3033	7.8	6.8	319
3034	3.0	2.4	356
4002	4.3	4.0	454
4006	3.5	3.1	495
4010	2.7	2.6	554
4012	4.7	4.4	303
4018	3.7	3.4	573

ART, antiretroviral therapy; pVL, plasma viral load.

### 3.2 Treatment interruption with close follow‐up is safe

ATI was generally safe as no serious AEs were recorded. However, most participants (13/16; 81%) reported an AE, with a total of 32 AEs recorded. All AEs were mild or moderate in intensity and resolved by the end of the study period. No side effects specifically related to the leukapheresis were reported. Three AEs (in three participants, 18.8%) were deemed to be related to the treatment interruption, occurred around the time of viral rebound and resolved spontaneously (Table S2). These may have contributed to the decision to restart ART.

### 3.3 Rapid viral rebound following treatment interruption, complete resuppression of viraemia and normalization of reservoir measures after treatment restart

**Figure 2:**
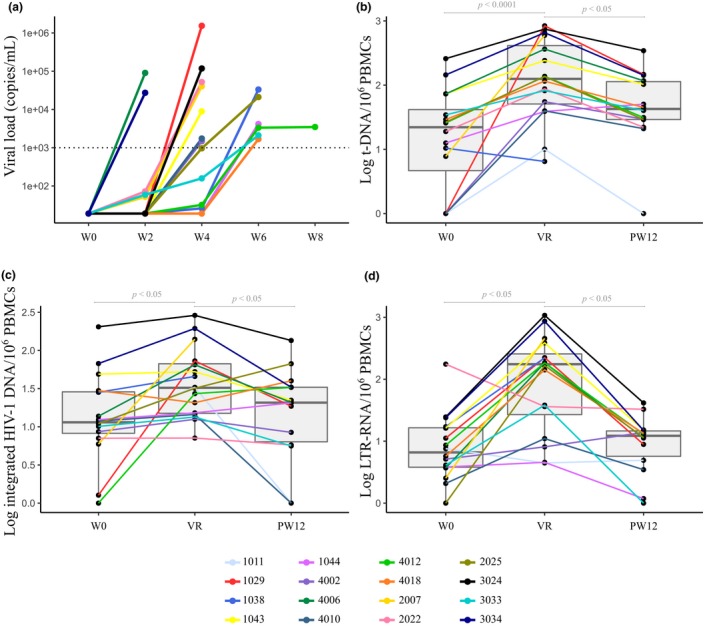
**(a)** Plasma viral rebound dynamics after ATI until ART restart in 16 selected patients with very small viral reservoir, **(b, c, d)** dynamics of total and integrated HIV‐1 DNA and LTR HIV‐1 CA‐RNA over time in stage 2 patients. *p*‐Values are from Friedman Rank Sum tests and *post‐hoc* Nemenyi test. ART, antiretroviral therapy; ATI, analytical treatment interruption; LTR, long terminal repeat; PBMC, peripheral blood mononuclear cell; PW12: 12 weeks after restart ART; VR, timepoint at viral rebound just before restart ART, W, weeks; W0, timepoint at stop ART.

All participants experienced viral rebound between two and eight weeks post ATI (Figures 2a and S1). All but one participant resumed ART immediately after reaching viral rebound criteria. Participant 4012 reached rebound criteria by week 8 but maintained pVLs < 3500 copies/mL until week 11, when ART was restarted. By 12 weeks after ART restart, all participants in follow‐up suppressed viraemia again below 50 copies/mL. Two participants were lost to follow‐up after treatment restart. One restarted treatment shortly after the viral rebound and returned to follow‐up 11 months later with an undetectable viral load. The other one had had an undetectable viral load four weeks after restart but moved to another country afterwards.

Total and integrated DNA and LTR‐RNA were determined before and during ATI as well as after restart of ART on bulk PBMCs (Figures 2b,c,d and S1). At the time‐point before ART resumption, total and integrated DNA as well as LTR‐RNA were significantly higher as compared to baseline (*p* < 0.0001, <0.05 and <0.05 respectively). By week 12 post ART resumption, integrated DNA and LTR‐RNA normalized to baseline levels, while t‐DNA remained slightly but non‐significantly higher than baseline (*p* > 0.05).

### 3.4 TTVR is not associated with residual viraemia, *in vitro* viral release, qVOA and LTR‐RNA levels

Clinical parameters such as HIV risk group, treatment regimen, time on treatment, nadir and current CD4+ T‐cell count were not associated with TTVR. To further characterize the 16 strictly selected stage 2 participants, we measured additional viral parameters at baseline, including residual viraemia (usVL), *in vitro* viral outgrowth, *in vitro* viral release and LTR‐RNA levels in CD4+ T cells before ATI.

**Figure 3:**
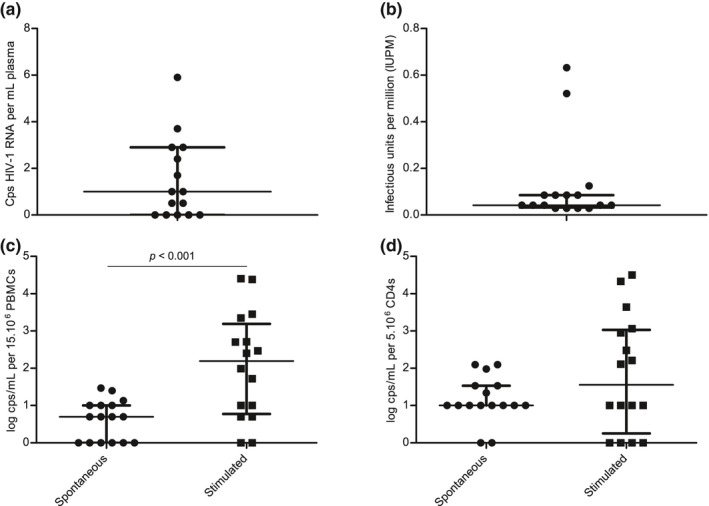
Additional viral parameters before ATI. **(a)** Ultra‐sensitive plasma viral load (in HIV‐1 RNA copies/mL). **(b)** Replication competent fraction (in infectious units per million CD4+ T cells, IUPM). **(c, d)** Viral release from 15 × 10^6^ PBMCs (c) and 5 × 10^6^ CD4+ T cells (d), either spontaneous or after stimulation with PMA + ionomycin (in log copies/mL). *p* Values are from Wilcoxon matched‐pairs signed‐rank test. ATI, analytical treatment interruption; PBMC, peripheral blood mononuclear cell.

Only one of the selected participants had a pVL > 5 copies/mL (5.9 copies/mL) while on ART; nine had a detectable pVL < 5 copies/mL and five had undetectable pVLs (Figure 3a). Plasma from one participant (2025) was not available for testing. There was no association between residual viraemia and TTVR (Figure S5).

In the qVOA assay, very low replication competent fractions were observed in the participants, with a median of 0.042 infectious units per million cells [IQR: 0.032 to 0.085] (Figure 3b). Again, there was no association between the size of the replication competent fraction and TTVR. To further distinguish the selected participants based on transcriptional activity, LTR‐RNA measurements were repeated at baseline in enriched CD4+ T cells instead of bulk PBMCs. As opposed to US‐RNA in PBMCs, LTR‐RNA in CD4+ T cells was always detectable and ranged from 6.5 to 744.6 copies per million CD4+ T cells (median: 105.4 copies) but was not associated with TTVR (Figure S2).

Finally, the recently described VRA was performed. Spontaneous viral release from PBMCs was significantly lower in stage 2 participants as compared to a selection of 12 stage 1 participants with the highest t‐DNA levels (median 441 copies per million PBMCs) (Figure S3). Significantly larger amounts of virus were spontaneously released per million CD4+ T cells as compared to PBMCs (median 1.00 vs. 0.22 log copies/mL, *p* < 0.05) as well as with stimulated versus non‐stimulated PBMCs (median 1.77 vs. 0.22 log copies/mL, *p* < 0.001) (Figure 3c,d). Nevertheless, TTVR was not associated with spontaneous or stimulated viral release in PBMCs or CD4+ T cells. Interestingly, at baseline, the level of LTR‐RNA in CD4+ T cells significantly correlated with the amount of viral RNA released in the supernatant of PBMC or CD4+ T cells with or without stimulation as measured in the VRA assay (Figure S4).

**Figure 4:**
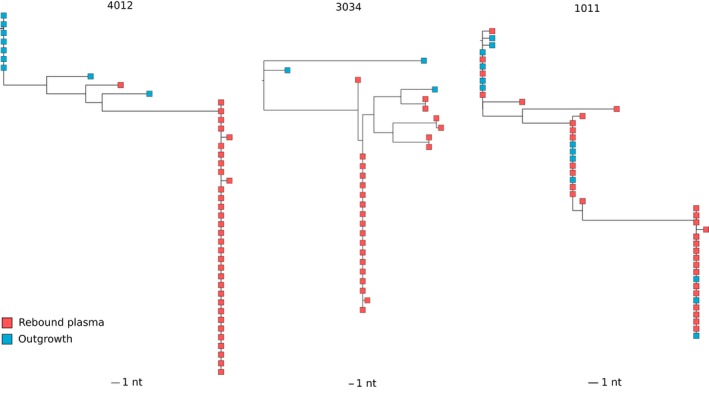
Maximum likelihood phylogenetic trees of V1‐V3 *env* sequences from rebound plasma and positive quantitative viral outgrowth assay wells (outgrowth) for participants 4012, 3034 and 1011.

Clustering of rebound sequences and *in vitro* viral outgrowth sequences indicates close relatedness and potential value of qVOA To examine the relationship between *in vivo* viral rebound and *in vitro* viral outgrowth viruses, V1‐V3 *env* sequences obtained by SGA were compared between plasma rebound viruses and qVOA viruses at baseline in the three participants with more than two positive qVOA wells (1011, 4012 and 3033). A total of 125 plasma rebound sequences were compared to 24 sequences obtained from qVOA. Rebound and qVOA sequences phylogenetically clustered together for each participant (Figure S5). When analysing the individual maximum likelihood phylogenetic tree (Figure 4), no identical sequences were found between rebound and qVOA viruses for two participants (4012 and 3034), although all sequences intermingled and were phylogenetically closely related. In contrast, in participant 1011, all 12 qVOA viruses were also found in the rebound plasma and clustered together in three distinct but closely related clusters.

To estimate the probability that identical viruses in the V1‐V3 *env* region truly represent viral clones, the clonal prediction score described by Laskey *et al*. 2016 was calculated 27. For the V1‐V3 *env* region, this score is 88.95 (SD 15.605), giving 89% chance that two or more viruses are identical over the entire genome if they are identical in the V1‐V3 *env* region, based on 45 publicly available unique detectable full‐genome sequences derived from qVOAs and rebound plasma.

## 4 Discussion

In this study, we hypothesized that ART treated HIV‐infected individuals selected for both very low t‐DNA and US‐RNA, would have a delayed viral rebound after interrupting ART. However, all 16 selected participants rebounded within eight weeks after ATI, which is similar to what has been observed in other ATI studies using less stringent criteria 28, 29. In line with most ATI studies, however, no major AEs were recorded and all reservoir measures returned to baseline after treatment restart 5, 28, 30.

It is important to consider that most patients (93%) with below‐threshold levels of t‐DNA also had below‐threshold levels of US‐RNA. Consequently, participants undergoing ATI were primarily selected on reservoir size and less so on transcriptional activity. The rapid viral rebound observed in this study is therefore consistent with a recently published ATI study which included patients selected on only small reservoir size, and observed viral rebound in nine of ten patients within four weeks (eight patients) and 12 weeks (one patient) while just one patient maintained pVL below 400 copies/mL for 56 weeks 28.

Exploratory objectives included an association analysis between TTVR and demographic, clinical and biological parameters. No correlations were observed between these parameters and TTVR. In view of these results, we decided *post‐hoc* to quantify on‐going viral transcription by LTR‐RNA at baseline as a measure of transcription initiation as opposed to transcription completion (US‐RNA). As recently observed by Yukl *et al.* 21, LTR‐RNA levels were much higher than US‐RNA levels (Figure S2). Interestingly, LTR‐RNA levels correlated significantly with the amount of virus released in *in vitro* culture (VRA) but were not associated to TTVR. While the clinical relevance of these markers of early transcription is unclear, they are far more sensitive than US‐RNA. Future ATI studies could therefore consider using such markers to select potential study participants.

To investigate the origin of viral rebound, we compared qVOA outgrowth viruses with *in vivo* plasma rebound viruses using SGA. Exact matches between qVOA and rebound clones have been reported only very rarely 31, 32. In these three participants, we observe intermingling between the viral sequences obtained from qVOA and plasma at viral rebound, confirming their close phylogenetic relatedness. Interestingly, in one participant, three distinct viral sequence expansions from the qVOA had exact matches with sequence expansions observed in the V1‐V3 *env* rebound sequences. To further investigate whether viral outgrowth assays have a predictive value for viral rebound in particular cases more research needs to be done, including more participants.

This study had a number of limitations. First, we achieved only half of the sample size which we aimed for at the onset of the study (16 instead of 32 stage two participants). Assuming a proportion of 5% to 10% of PTCs in our participants, this smaller sample size increased the possibility of not finding any PTC from 5% to 18%. Second, all participants experienced viral rebound within a relatively short time frame, making differences in TTVR very small (0 to 6 weeks) and correlations unlikely, considering the small sample size. Third, participants were monitored only every other week. More frequent sampling (e.g. twice weekly) could have allowed a more refined analysis of rebound kinetics and more powerful correlation analyses. A fourth limitation, inherent to all recent HIV ATI studies, is that ART was generally restarted immediately or shortly after meeting the stringent viral rebound criteria. This may have prevented the detection of viral control after a transient viral rebound, as is observed in a large subset of PTCs as late as 24 weeks after ATI 6. Indeed, one participant experienced viral rebound after eight weeks but maintained pVLs below 3500 copies/mL for 11 weeks, after which the participant decided to restart therapy. Considering the good safety observed in recent ATI studies 24, and the lower probability of missing PTCs 6, future trials might consider adopting higher treatment restart thresholds. These advantages have to be weighed against the risk of exposing participants and their partners to longer periods of detectable viral loads and subsequent risk of transmission 33. Concerning the comparison between qVOA and rebound sequences, SGS was limited to the highly variable but relatively small V1‐V3 sub‐genomic region of *env*. While longer length SGA sequencing from positive VOA wells is feasible, this may prove to be more challenging for rebound plasma virus. Furthermore, sequencing the entire *env* gene would increase the clonal prediction score only slightly. The clonal prediction score of this amplicon is 89%, leaving an 11% chance that the matching viruses differ in one or more nucleotides somewhere else in the genome. However, this prediction score was calculated based on a limited database of 45 unique detectable sequences and therefore has a high standard deviation, making it difficult to interpret this score.

There remains quite some discussion about the value of qVOA when investigating the HIV reservoir, due to rather poor correlations with rebound sequences as described here and by others 34, 35, 36. One potential explanation is that qVOA is limited to the blood compartment and does not take into account rebound coming from other anatomical reservoirs. As shown by De Scheerder *et al*. rebound is heterogeneous and can be fuelled by any infected cell in any anatomical reservoir, making it difficult to have an *in vitro* proxy 37. Another potential explanation is the dynamic nature of the viral reservoir, which implicates that sequences between different time points and the observed clones might wax and wane 36, 38.

## 5 Conclusions

Although HIV‐1 reservoir markers such as t‐DNA and US‐RNA have been correlated with PTC status, we show here that their predictive power is too weak to prospectively identify PTCs from the general patient population on ART. PTC status most likely depends not only on the size, transcriptional activity and replication competence of the viral reservoir in the peripheral blood, but also on reservoirs elsewhere and the immune activity in the body. Developing reliable and easy assays to evaluate whole body parameters of viral activity and immune control remains a major challenge for HIV cure research.

## Competing interests

The authors declare no conflicts of interest.

## Authors' contributions

EF, GV, LV, SDW, SDA, JA and WDS conceptualized and designed the study. MC, PP, GV, EF and NH wrote the leukapheresis standard operating procedure (SOP) to be used in all centres. PP and GV wrote the laboratory analytical plan and all study specific SOPs. PP, SR, BC, WDS and MDS were in charge of setting up and performing the various viral and immunological assays and the processing of the resulting data. CN, EF, LV, SDA and SDW were in charge of the clinical follow‐up of included participants. NH managed the study and ensured adherence to study protocols. AT designed the statistical analysis plan and performed all statistical analyses. All authors were involved in the interpretation of the study results. PP and SR drafted the first version of the manuscript. All authors were involved in the revision and final approval of the version of the manuscript to be published.

## Abbreviations

AE, adverse event; ART, antiretroviral therapy; ATI, analytical treatment interruption; ddPCR, droplet digital PCR; EDTA, ethylenediaminetetraacetic acid; FCS, fetal calf serum; IQR, inter quartile range; ITM, Institute of Tropical Medicine; LTR, long terminal repeat; PBMC, peripheral blood mononuclear cell; PTC, post treatment control; pVL, plasma viral load; PW12, post week 12; qVOA, quantitative viral outgrowth assay; RPMI, Roswell Park Memorial Institute; SGA, single genome analysis; t‐DNA, total HIV DNA; TTVR, time to viral rebound; UMC, University Medical Center; US‐RNA, unspliced cell‐associated HIV RNA; usVL, ultra‐sensitive viral load; UZB, Universitair Ziekenhuis Brussel; UZG, Universitair Ziekenhuis Gent; VRA, viral release assay.

## Supporting information


**Figure S1.** Detailed viral parameters after analytical treatment interruption.
**Figure S2.** Transcriptional activity at baseline.
**Figure S3.** Viral release.
**Figure S4.** Transcriptional activity versus viral release.
**Figure S5.** Viremia versus time to viral rebound.
**Figure S6.** Single genome analysis patient clusters.
**Table S1.** Characteristics of study participants.
**Table S2.** Adverse events.
**Table S3.** Technical specifications of polymerase chain reaction assays.
**Methods S1.** Cell‐associated HIV‐1 DNA and RNA measurements at screening.
**Methods S2.** Quantitative viral outgrowth assay.
