# Supplementary material for: Rapid viral rebound after analytical treatment interruption in patients with very small HIV reservoir and minimal on‐going viral transcription
Source: J Int AIDS Soc. 2020 Feb 27;23(2):e25453. doi: 10.1002/jia2.25453 (PMC7046528; doi:10.1002/jia2.25453)
Supplement: Supplementary file 1 — Figure S1. Detailed viral parameters after analytical treatment interruption. Figure S2. Transcriptional activity at baseline. Figure S3. Viral release. Figure S4. Transcriptional activity versus viral release. Figure S5. Viremia versus time to viral rebound. Figure S6. Single genome analysis patient clusters. Table S1. Characteristics of study participants. Table S2. Adverse events. Table S3. Technical specifications of polymerase chain reaction assays. Methods S1. Cell‐associated HIV‐1 DNA and RNA measurements at screening. Methods S2. Quantitative viral outgrowth assay. [file JIA2-23-e25453-s001.docx]

**
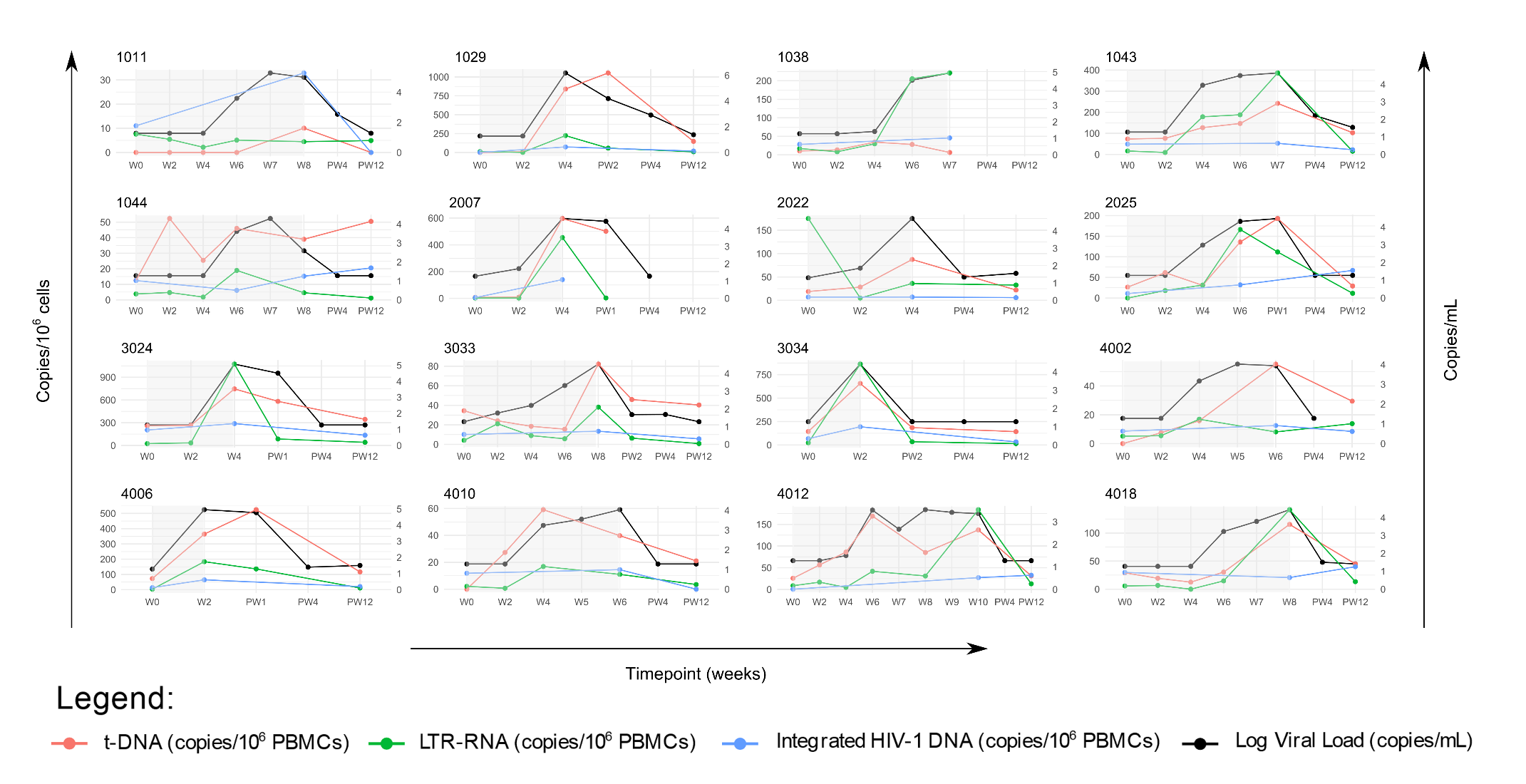
S1 Figure**. **Detailed viral parameters after ATI.** Individual dynamics of total and integrated HIV-1 DNA, LTR HIV-1 CA-RNA (copies/10^6^ cells) and viral load (copies/mL) over time in all stage 2 patients. The shaded area represents the period of treatment interruption, the white area represents time points under anti-retroviral therapy. (W: week after therapy interruption; PW: week post treatment restart)


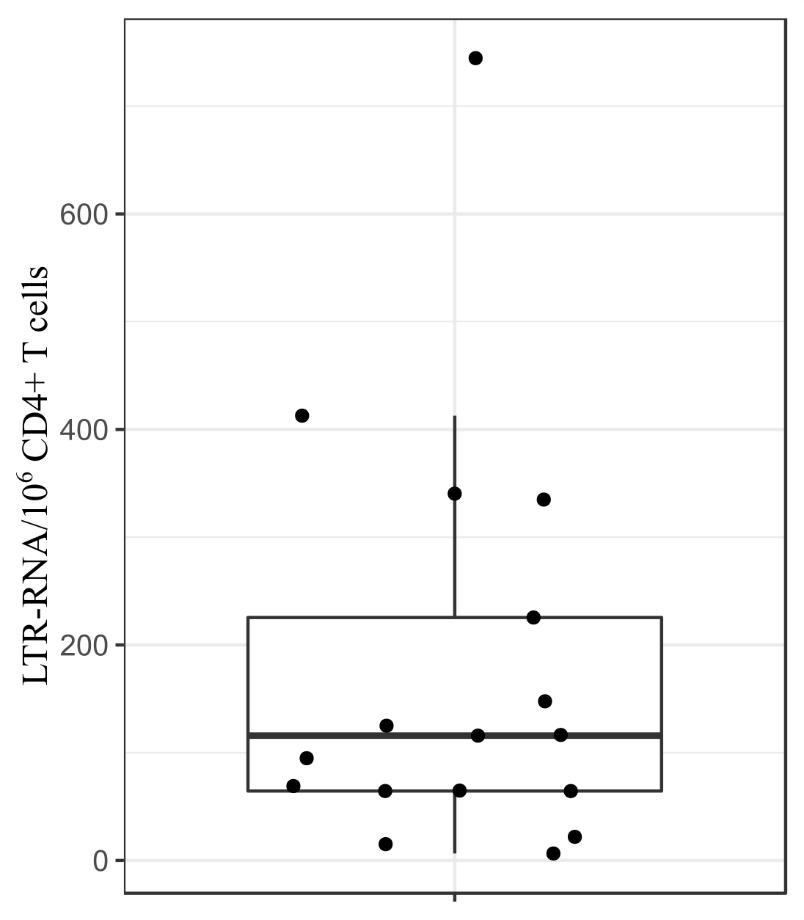


**S2 Figure. Transcriptional activity at baseline.** Cell-associated HIV-1 LTR-RNA quantification in CD4+ T cells in stage 2 patients at baseline.

**
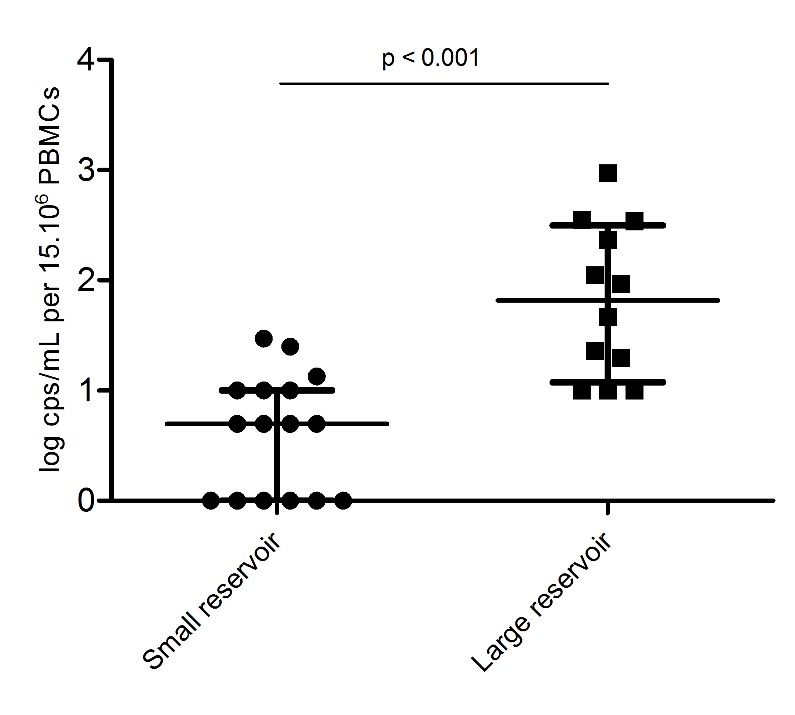
**

**S3 Figure. Viral release.** Spontaneous viral release from 15x10^6^ PBMCs before ATI without stimulation in patients with a small and large viral reservoir (in log copies/mL). P-value is from Mann-Whitney test.

**S4 Figure**. **Transcriptional activity versus viral release**. Correlation analysis between HIV-1 LTR-RNA and viral release assay. Spearman correlation coefficients were calculated and Locally Weighted Scatterplot Smoothing (LOWESS) curves are depicted.


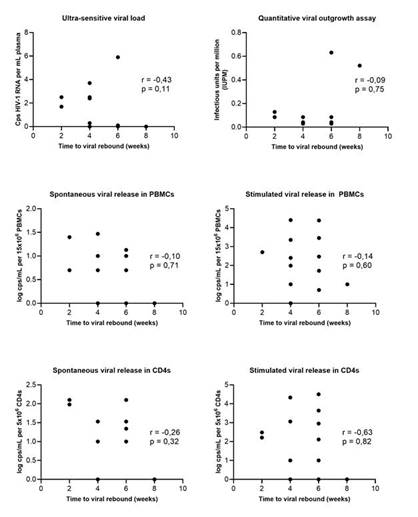


**S5 Figure: viremia versus time to viral rebound (TTVR)e**. Correlation analysis between various measurement of viremie and TTVR. Spearman correlation coefficients were calculated.


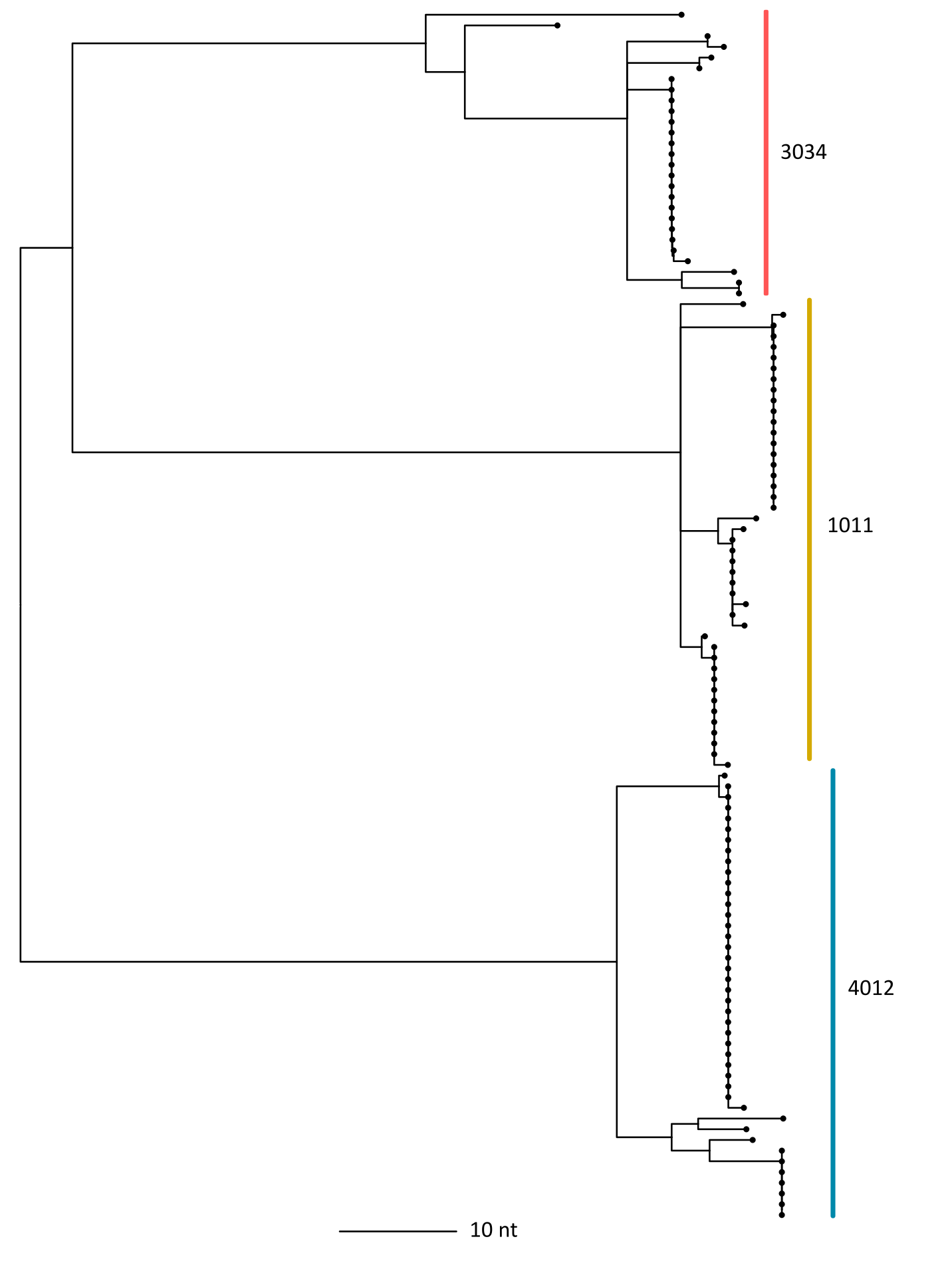


**S5 Figure. Single genome analysis patient clusters.** Neighbor-joining phylogenetic tree with all V1-V3 env sequences obtained from participants 3034, 1011 and 4012. Distinct clustering shows no relation between V1-V3 env sequences from different participants.

**S1 Table. Characteristics of study participants.**

|  | Enrolled in stage 1  (n=114) | Non eligible for stage 2  (n=76) | Eligible for stage 2  (n=38) | Enrolled in stage 2  (n=16) |
| --- | --- | --- | --- | --- |
| Age (years) | 45·5 (38·0 - 52·0) | 44·5 (38·0 - 52·0) | 46·0 (38·0 - 54·0) | 43·5 (38·0 - 54·0) |
| Female sex | 6 (5·3%) | 4 (5·3%) | 2 (5·3%) | 1 (6·3%) |
| Ethnicity |  |  |  |  |
| White | 111 (97·4%) | 73 (96·1%) | 38 (100%) | 16 (100%) |
| Other | 3 (2·6%) | 3 (3·9%) | 0 | 0 |
| HIV risk group |  |  |  |  |
| Men who have sex with men | 95 (83·3%) | 64 (84·2%) | 31 (81·6%) | 12 (75·0%) |
| Heterosexuals | 14 (12·3%) | 8 (10·5%) | 6 (15·8%) | 3 (18·8%) |
| Unknown | 4 (3·5%) | 3 (3·9%) | 1 (2·6%) | 1 (6·3%) |
| Transfusion, non-hemophilia related | 1 (0·9%) | 1 (1·3%) | 0 | 0 |
| Time since HIV diagnosis (years) | 5·9 (3·9 - 8·4) | 6·1 (4·0 - 9·5) | 5·1 (3·2 - 8·1) | 3·9 (2·8 - 6·3) |
| Time on ART (years) | 4·3 (3·0 - 6·0) | 4·3 (3·1 - 5·9) | 4·3 (2·9 - 6·6) | 4·0 (2·9 - 6·2) |
| Last used regimen |  |  |  |  |
| II + NRTI | 49 (43·0%) | 31 (40·8%) | 18 (47·4%) | 11 (68·8%) |
| NNRTI + NRTI | 51 (44·7%) | 38 (50·0%) | 13 (3·.2%) | 4 (25·0%) |
| PI + NRTI | 4 (3·5%) | 2 (2·6%) | 2 (5·3%) | 0 |
| Other | 10 (8·8%) | 5 (6·6%) | 5 (13·2%) | 1 (6·3%) |
| HIV subtype B infected | 106 (93·0%) | 71 (93·4%) | 36 (92·3%) | 15 (93·8%) |
| Nadir CD4 (cells/µL) | 379·0 (338·0 - 482·0) | 368·0 (330·0 - 476·0) | 426·5 (350·0 - 506·0) | 440·5 (342·0 - 500·5) |
| CD4 count (cells/µL) | 733·0 (645·0 - 891·0) | 733·0 (647·0 - 903·5) | 735·0 (644·0 - 854·0) | 758·0 (679·0 - 845·0) |

Data are median (IQR) or n (%). ART = antiretroviral therapy. II = integrase inhibitor. NRTI=nucleoside reverse transcriptase inhibitor. NNRTI=non-nucleoside reverse transcriptase inhibitor. PI=protease inhibitor.

**S2 Table. Adverse events (AE).**

| All adverse events | n (%) |
| --- | --- |
|  |  |
| Any AE | 13 (81·3) |
| Gastrointestinal disorders | 3 (18·8) |
| Abdominal pain | 1 (6·3) |
| Soft faeces | 1 (6·3) |
| Stomatitis | 1 (6·3) |
| Vomiting | 1 (6·3) |
| General disorders and administration site conditions | 7 (43·8) |
| Fatigue | 3 (18·8) |
| Influenza like illness | 4 (25·0) |
| Infections and infestations | 8 (50·0) |
| Bronchitis | 1 (6·3) |
| Ear infection | 1 (6·3) |
| Fungal infection | 1 (6·3) |
| Influenza | 1 (6·3) |
| Nasopharyngitis | 4 (25·0) |
| Oral herpes | 1 (6·3) |
| Pyelonephritis | 1 (6·3) |
| Rhinitis | 1 (6·3) |
| Tracheitis | 1 (6·3) |
| Metabolism and nutrition disorders | 1 (6·3) |
| Vitamin D deficiency | 1 (6·3) |
| Musculoskeletal and connective tissue disorders | 3 (18·8) |
| Arthralgia | 1 (6·3) |
| Back pain | 1 (6·3) |
| Fasciitis | 1 (6·3) |
| Psychiatric disorders | 1 (6·3) |
| Insomnia | 1 (6·3) |
| Renal and urinary disorders | 1 (6·3) |
| Dysuria | 1 (6·3) |
| Respiratory, thoracic and mediastinal disorders | 3 (18·8) |
| Cough | 2 (12·5) |
| Oropharyngeal pain | 1 (6·3) |
|  |  |
| All adverse events related to treatment interruption | |
|  |  |
| Any AE | 3 (18·8) |
| General disorders and administration site conditions | 2 (12·5) |
| Fatigue | 1 ( 6·3) |
| Influenza like illness | 1 ( 6·3) |
| Respiratory, thoracic and mediastinal disorders | 1 ( 6·3) |
| Oropharyngeal pain | 1 ( 6·3) |

**S3 Table**. Technical specifications of polymerase chain reaction assays.

| **Assay** | **Location** | **Primer** | **Specifications** | **Label** | **Temperature** | **Sequence** |
| --- | --- | --- | --- | --- | --- | --- |
| t-DNA | HIV RU5 | Forward |  |  |  | 5’-TTAAGCCTCAATAAAGCTTGCC-3’ |
|  | HIV LTR-Gag inter | Reverse |  |  |  | 5’-GTTCGGGCGCCACTGCTAGA-3’ |
|  | HIV RU5 | Probe |  | FAM | 58°C | 5'-CCAGAGTCACACAACAGACGGGCACA-3' |
| RPP30 | human RPP30 gene | Forward |  |  |  | 5'-AGATTTGGACCTGCGAGCG-3' |
|  | human RPP30 gene | Reverse |  |  |  | 5'-GAGCGGCTGTCTCCACAAGT-3' |
|  | human RPP30 gene | Probe |  | HEX | 58°C | 5'-TTCTGACCTGAAGGCTCTGCGCG-3' |
| Integrated HIV-1 DNA | human Alu | Forward | outer PCR |  |  | 5’-GCCTCCCAAAGTGCTGGGATTACAG-3’ |
|  | HIV LTR-Gag inter | Reverse | outer PCR |  |  | 5’-GTTCGGGCGCCACTGCTAGA-3’ |
|  | HIV LTR | Forward | inner PCR |  |  | 5’-GCCTCAATAAAGCTTGCC-3' |
|  | HIV LTR | Reverse | inner PCR |  |  | 5’-GGCGCCACTGCTAGAGATTTT-3' |
|  | HIV LTR-Gag inter | Probe | inner PCR | MGB/FAM | 60°C | 5’-AAGTRGTGTGTGCCC-3' |
| US-RNA | HIV Gag | Forward |  |  |  | 5' -CATGTTTTCAGCATTATCAGAAGGA-3' |
| HIV-1 subtype B |  | Reverse |  |  |  | 5'-TGCTTGATGTCCCCCCACT-3' |
|  |  | Probe |  | FAM | 58°C | 5'-CCACCCCACAAGATTTAAACACCATGCTAA-Q 3' |
| US-RNA | HIV Pol | Forward |  |  |  | 5'-TACAGTGCAGGGGAAAGAATA-3’ |
| HIV-1 subtype non-B |  | Reverse |  |  |  | 5'-CTGCCCCTTCACCTTTCC-3’ |
|  |  | Probe |  | FAM | 58°C | 5'-TTTCGGGTTTATTACAGGGACAGCAG-3’ |
| LTR-RNA | HIV LTR | Forward |  |  |  | 5’-GCCTCAATAAAGCTTGCC-3' |
|  | HIV LTR-Gag inter | Reverse |  |  |  | 5’-GGCGCCACTGCTAGAGATTTT-3' |
|  | HIV LTR | Probe |  | MGB/FAM | 56°C | 5’-AAGTRGTGTGTGCCC-3' |

**S1 Methods. Cell-associated HIV-1 DNA and RNA measurements at screening**

t-DNA and US-RNA targeting the gag/pol region were measured at screening by droplet digital PCR (ddPCR) as previously described (1). Briefly, genomic DNA (gDNA) was extracted from 10 million PBMCs by the DNeasy Blood & Tissue kit (Qiagen, Hilden, Germany) using manufacturer’s protocol with an additional step of heating 75µl elution buffer on the column at 56°C for 10min. Before amplification, 8.65µl of genomic DNA was restricted by EcoRI in a total volume of 10µl restriction digest. RNA was extracted from a separate vial of 10 million PBMCs by Innuprep RNA kit in 30µl elution buffer (Westburg, Leusden, The Netherlands). Extraction yields were verified by Qubit 3.0 Fluorometer (Thermo Fisher Scientific, Geel, Belgium). A total of 1,000ng RNA was reverse transcribed to cDNA by qScript cDNA SuperMix (Quantabio, Beverly, MA, USA) and used for further quantification.

t-DNA and US-RNA were measured by ddPCR by adding respectively 2µl and 4µl in triplicates to ddPCR mix containing 10µl 2x ddPCR Supermix for Probes, respectively 800nM/500nM primers and 300 nM probe (Integrated DNA Technologies (IDT), Belgium). Two sets of primers and probe were used for US-RNA depending on the HIV-1 subtype to ensure adequate pick-up of HIV-1 non-B subtypes (primers and probes are depicted in S2 Table). PCR amplification was performed with an initial denaturation step of 5min at 95°C followed by 40 cycles of a denaturation step for 30sec at 95°C and annealing/elongation for 1min at 58°C. Droplets were read by QX200 droplet reader (Bio-Rad) and analyzed using ddpcRquant software (2).

t-DNA was normalized by measuring the reference gene RPP30 in duplicate by ddPCR and expressed per million PBMCs. US-RNA was normalized by dividing copies HIV RNA by the geometric mean of three reference genes per patient, B2M, YMHAZ and HMBS, measured with LightCycler480 SYBR Green I Master mix (Roche Applied Science, Belgium) and expressed per million cells by normalizing to the theoretical number of cells per µl RNA.

**Cell-associated HIV-1 DNA and RNA measurements during stage 2**

In stage 2 participants, t-DNA and cell-associated LTR HIV-1 RNA (LTR-RNA) were measured in PBMCs at baseline, on weeks 2, 4, 6, 8, 12, 16, 20, 24, 32, 40 and 48 post-ATI and week 12 post-ART restart (PW12) (Figure 1A), integrated HIV-1 DNA at baseline, before restart ART and PW12, and LTR-RNA in CD4+ T cells at baseline. LTR-RNA measures not only the unspliced HIV-1 RNA but targets all HIV transcripts elongated beyond 5’-LTR region (3, 4). For this particular assay, RNA was reverse transcribed to cDNA by qScript XLT cDNA SuperMix (Quantabio, MA, USA) allowing a maximum input of 2,000ng instead of 1,000ng. To increase the sensitivity of LTR-RNA quantification at baseline a second measurement was performed on enriched CD4+ T cells: CD4+ T cells were isolated from PBMCs at baseline using EasySep Human CD4+ T Cell Isolation Kit (Stemcell technologies, Vancouver, Canada). ddPCR for HIV DNA and HIV RNA quantification was performed as described above.

Integrated HIV-1 DNA was measured by repetitive sampling of Alu-HIV PCR based on Poisson statistics as described previously (5). Briefly, 40 replicates were analyzed by a nested qPCR with a first round targeting human Alu gene and HIV-1 LTR, followed by a second round targeting HIV LTR (primers and probe in S2 Table). To correct for background noise, 20 replicates without the forward Alu primer were run in parallel. The input of genomic DNA was set at 2 copies of total HIV-1 DNA per replicate. The number of copies integrated HIV-1 DNA was calculated based on the number of positive wells with Poisson statistics and normalized per million PBMCs by reference gene RPP30 quantification by ddPCR.

## **S2 Methods. Quantitative viral outgrowth assay (qVOA)**

This assay was based on Laird et al. with some modifications (6). In short, 24 x 10^6^ CD4+ T cells, enriched from PBMCs using positive selection (Miltenyi, Bergisch Gladbach, Germany), were plated on day 0 in two 24 well plates at 5 x 10^5^ cells/well together with 5 x 10^6^ γ-irradiated allogeneic PBMCs from uninfected donors in 2mL RPMI (Westburg, Leusden, The Netherlands) containing 15% FCS (Life Technologies, Ghent, Belgium), 200U/mL interleukin-2 (IL-2, Gentaur, Kampenhout, Belgium) and 1µg/mL phytohaemagglutinin PHA (Thermo Scientific, Merelbeke, Belgium). Donor PBMCs were isolated from buffy coats, obtained from the Red Cross Transfusion Center in Mechelen, Belgium. On day 1, the cells were washed twice and 10^5^ MOLT-4/CCR5 cells (NIH AIDS Reagent Program) were added per well. IL-2 containing medium was refreshed twice per week and supernatant of each well was tested for infectious HIV-1 after one, two, three and four weeks of culture with the TZM-bl assay (NIH AIDS Reagent Program) (7). The frequency of replication-competent latently infected cells was calculated by a maximum likelihood method, as described previously, and is expressed as infectious units per million cells (IUPM) (8).

**References**

1. Kiselinova M, Geretti AM, Malatinkova E, Vervisch K, Beloukas A, Messiaen P, et al. HIV-1 RNA and HIV-1 DNA persistence during suppressive ART with PI-based or nevirapine-based regimens. The Journal of antimicrobial chemotherapy. 2015;70(12):3311-6.

2. Trypsteen W, Vynck M, De Neve J, Bonczkowski P, Kiselinova M, Malatinkova E, et al. ddpcRquant: threshold determination for single channel droplet digital PCR experiments. Anal Bioanal Chem. 2015;407(19):5827-34.

3. Schvachsa N, Turk G, Burgard M, Dilernia D, Carobene M, Pippo M, et al. Examination of real-time PCR for HIV-1 RNA and DNA quantitation in patients infected with HIV-1 BF intersubtype recombinant variants. Journal of virological methods. 2007;140(1-2):222-7.

4. Yukl SA, Kaiser P, Kim P, Telwatte S, Joshi SK, Vu M, et al. HIV latency in isolated patient CD4(+) T cells may be due to blocks in HIV transcriptional elongation, completion, and splicing. Science translational medicine. 2018;10(430).

5. De Spiegelaere W, Malatinkova E, Lynch L, Van Nieuwerburgh F, Messiaen P, O'Doherty U, et al. Quantification of integrated HIV DNA by repetitive-sampling Alu-HIV PCR on the basis of poisson statistics. Clinical chemistry. 2014;60(6):886-95.

6. Laird GM, Rosenbloom DI, Lai J, Siliciano RF, Siliciano JD. Measuring the Frequency of Latent HIV-1 in Resting CD4(+) T Cells Using a Limiting Dilution Coculture Assay. Methods in molecular biology. 2016;1354:239-53.

7. Platt EJ, Wehrly K, Kuhmann SE, Chesebro B, Kabat D. Effects of CCR5 and CD4 cell surface concentrations on infections by macrophagetropic isolates of human immunodeficiency virus type 1. Journal of virology. 1998;72(4):2855-64.

8. Siliciano JD, Siliciano RF. Enhanced culture assay for detection and quantitation of latently infected, resting CD4+ T-cells carrying replication-competent virus in HIV-1-infected individuals. Methods in molecular biology. 2005;304:3-15.
